# The Interaction of Plasma Sialylated and Desialylated Lipoproteins with Collagen from the Intima and Media of Uninvolved and Atherosclerotic Human Aorta

**DOI:** 10.1155/2011/254267

**Published:** 2011-11-17

**Authors:** Igor A. Sobenin, Igor V. Suprun, Vasiliy P. Karagodin, Alexander S. Feoktistov, Alexandra A. Melnichenko, Alexander N. Orekhov

**Affiliations:** ^1^Laboratory of Cellular Mechanisms of Atherogenesis, Institute of General Pathology and Pathophysiology, 8 Baltiyskaya Street, 125315 Moscow, Russia; ^2^Laboratory of Cellular Mechanisms of Atheroscleroris, Institute of Experimental Cardiology and Laboratory of Medical Genetics, A.N. Myasnikov Institute of Clinical Cardiology, Cardiology Research Center, 15a 3rd Cherepkovskaya Street, 121552 Moscow, Russia; ^3^Institute for Atherosclerosis Research, Skolkovo Innovative Center, 121355 Moscow, Russia; ^4^Department of Human and Animal Physiology, Faculty of Biology, M.V. Lomonosov Moscow State University, 1-12 Leninskie Gory, 119991 Moscow, Russia

## Abstract

We have evaluated the binding of sialylated and desialylated lipoproteins to collagen isolated from the proteoglycan and musculoelastic layers of intima and media of uninvolved human aorta and atherosclerotic lesions. Comparing various collagen preparations from the uninvolved intima-media, the binding of sialylated apoB-containing lipoproteins was best to collagen from the intimal PG-rich layer. Binding of sialylated apoB-containing lipoproteins to collagen from this layer of fatty streak and fibroatheroma was 1.4- and 3.1-fold lower, respectively, in comparison with normal intima. Desialylated VLDL versus sialylated one exhibited a greater binding (1.4- to 3.0-fold) to all the collagen preparations examined. Desialylated IDL and LDL showed a higher binding than sialylated ones when collagen from the intimal layers of fibroatheroma was used. Binding of desialylated HDL to collagen from the intimal PG-rich layer of normal tissue, initial lesion, and fatty streak was 1.2- to 2.0-fold higher compared with sialylated HDL.

## 1. Introduction

Deposition of extracellular lipids on the components of the connective tissue matrix is one of the earliest and most pronounced manifestations of atherosclerotic lesion in human vessels. At present, data are available for the interaction of lipoproteins, above all of low-density lipoprotein (LDL), with elastin and proteoglycans (PGs) of the vessel wall [1–4]. Another main component of the extracellular matrix is collagen [5–7]. Although its role in the structural organization of the vascular wall and platelet aggregation has been conclusively demonstrated [8, 9], little is known concerning its interaction with blood lipoproteins that enter the arterial intima or its involvement in extracellular lipid accumulation in atherosclerotic arteries. Many investigators have reported a rise in the total collagen level in atherosclerotic lesions [10–12]. However, the data concerning the changes of collagen composition in the arterial intima during atherogenesis are contradictory [13–16]. In a number of other studies, LDL binding to different collagen types was examined [17–20]. Most of these studies included the use of collagen isolated from collagen-rich tissues of animals. Binding of LDL and other human blood lipoproteins to collagen isolated from various layers of the normal and atherosclerotic human aorta has not been studied so far.

Earlier, we identified circulating multiple-modified LDL. Unlike native LDL, modified LDL induced an accumulation of cholesterol and cholesteryl esters in smooth muscle cells cultured from the uninvolved human aortic intima [21–23]. The major characteristic feature of modified LDL was a low content of sialic acid, the terminal carbohydrate of the biantennary sugar chains in apoB and carbohydrate chains in gangliosides [24]. This property allowed us to develop a method for isolating desialylated particles from the total lipoprotein fraction using lectin chromatography on *Ricinus communis* agglutinin (RCA_120_) agarose. Modified, desialylated LDL was also characterized by a low level of neutral lipids, decreased content of phospholipids and fat-soluble vitamins, smaller size, higher density, increased electronegative charge, and altered tertiary apolipoprotein structure. Recently, using lectin chromatography, we have determined the presence of desialylated subfractions of very-low-density lipoprotein (VLDL) and intermediate-density lipoprotein (IDL) in human blood. Like desialylated LDL, desialylated VLDL and IDL induced cholesterol accumulation in the cells of unaffected human aortic intima. In this study, we have performed a quantitative comparative analysis of the binding of sialylated and desialylated VLDL, IDL, LDL, and high-density lipoprotein (HDL) to collagen isolated from two layers of the intima and the media taken from normal regions, initial lesions, fatty streaks, and fibroatheromas of human aorta.

## 2. Materials and Methods

### 2.1. Isolation of Collagen

Seven human thoracic aortas from men aged 35–55 years were taken aseptically within 1 to 3 h after sudden death from myocardial infarction. After mechanical separation of the adventitia, vessels were incised longitudinally, and the type of atherosclerotic lesion was determined according to the classification suggested by Stary [25]. Tissue of each type (normal or affected) was pooled. Intima was separated from media and divided mechanically into proteoglycan- (PG-) rich (elastic-hyperplastic) and musculoelastic layers. The accuracy of the procedure was verified by light microscopy [26]. The collagen was extracted from aortic tissue samples (0.2–2.5 g) using the Lansing procedure [27] modified by Noma et al. [1] and delipidated three times with chloroform/methanol (2 : 1, v/v), two times with acetone and then once with diethyl ether. A suspension of collagen (500 *μ*g/mL in PBS, pH = 7.2) was obtained by sonication in an ice-water bath for ten repetitive cycles for an overall period of 15 minutes.

### 2.2. Isolation of Lipoproteins

Blood was drawn from the ulnar vein of healthy subjects and patients with coronary atherosclerosis into plastic tubes containing EDTA (1 mg/mL blood). Blood cells were removed by two-step centrifugation at 3000 rpm for 10 min. Total VLDL, IDL, LDL, and HDL fractions were isolated by ultracentrifugation as described earlier [17]. Lipoproteins were dialyzed against 3 × 2000 volumes of 50 mM Tris-HCl, pH 7.0 at 4°C overnight, and sterilized by filtration (0.45 *μ*m). Lectin chromatography on *Ricinus communis* agglutinin (RCA_120_; Boehringer Mannheim GmbH, Mannheim, Germany) bound to agarose was used to separate the sialylated and desialylated lipoproteins [16]. Sialylated and desialylated VLDL, IDL, and LDL were iodinated according to Bilheimer et al. [28]. The specific activity of ^125^I-lipoprotein ranged from 55 to 250 cpm/ng protein. Sialylated and desialylated HDL was iodinated (42–102 cpm/ng protein) according to Markwell [29].

Sialylated lipoproteins were treated with 0.02 U/mL neuraminidase from *Arthrobacter ureafaciens* (Oxford GlycoSystems, Oxford, UK) at 37°C for 6 h.

### 2.3. Binding Studies

96-well microtiter plates (Nunc, Roskilde, Denmark) were coated with 100 *μ*L collagen per well at concentrations of 1–10 *μ*g/mL in PBS, pH = 7.2 by incubation at 4°C for 24 h. The wells were washed four times with PBS containing 0.05% Tween-20, and 100 *μ*L of PBS containing 2% bovine serum albumin (PBS-BSA) was added to the wells to block remaining nonspecific binding sites. After reaction at 20°C for 1 h, the wells were washed with PBS-BSA, and 100 *μ*L per well of iodinated lipoprotein (1–60 *μ*g protein/mL) was added and incubated at 37°C for 1–3 h. Following incubation, the plates were washed with PBS-BSA. Bound iodinated lipoprotein was released with 200 *μ*L of 0.1 N NaOH, and radioactivity was measured with RackGamma II gamma counter (Wallac OY, Turku, Finland).

### 2.4. Statistical Analysis

The significance of differences between group mean values was evaluated using a BMDP statistical program package [30].

## 3. Results

### 3.1. Binding of Sialylated and Desialylated VLDL to Collagen

Preliminary experiments showed that the binding of both sialylated and desialylated VLDL to collagen isolated from various layers of the uninvolved aortic intima-media did not depend on collagen concentration, used for coating the wells of microtiter plates, in the range of 1–10 *μ*g/mL (data not shown). Concentration curves and time curves of VLDL binding were linear over the lipoprotein concentration range of 1–30 *μ*g protein/mL and within a period of 1–3 h (not shown). Similar results were obtained with other lipoproteins. In all subsequent experiments, collagen concentration in the coating solution was 2 *μ*g/mL and lipoproteins were incubated in the collagen-coated wells at the concentration of 10 *μ*g protein/mL for 2 h.

Table 1 shows the data obtained with VLDL. Sialylated VLDL bound at a similar extent to collagen from the intimal PG-rich layer of uninvolved area and that of initial lesion. The amount of sialylated VLDL binding to collagen from the fatty streak and fibroatheroma was 3.1- and 2.3-fold lower, respectively, in comparison with normal tissue.

Between the two collagens of the uninvolved intima, 2.9-fold less sialylated VLDL was bound to collagen from the musculoelastic layer. The binding capacity for sialylated VLDL of collagen from the musculoelastic layer did not depend on the stage of lesion progression and was the same with normal and diseased tissue.

In a similar manner, collagen of media, regardless of whether it was isolated from the areas under unlesioned intima, initial lesions, fatty streaks, or fibroatheromas, had an identical binding capacity for sialylated VLDL. In addition, the extent of sialylated VLDL binding to collagen from the media was the same as to collagen from the musculoelastic layer of the intima.

With all collagen preparations, desialylated VLDL exhibited greater binding than did sialylated VLDL: the amount of bound desialylated VLDL was 1.4- to 3.0-fold above the amount of bound sialylated one.

### 3.2. Binding of Sialylated and Desialylated IDL to Collagen

Table 2 summarizes the results obtained with IDL. Sialylated IDL showed similar binding to collagen from the PG-rich layer of normal intima and that of the initial lesion. The binding of sialylated IDL to collagen from this layer of fatty streak and of fibroatheroma was 2.4- and 1.6-fold lower, respectively, compared with uninvolved areas.

We did not find any significant difference in binding of sialylated IDL by collagen between the intimal musculoelastic layer and medial layer, or between normal and affected regions of these layers.

When extracted from the PG-rich or musculoelastic layer of uninvolved intima, an initial lesion, or the fatty streak, collagen bound desialylated and sialylated IDL to a similar extent; however, when isolated from these layers of fibroatheroma, it exhibited an increased (1.3-fold) binding capacity for desialylated IDL. No significant difference in binding to collagen from the media underlying normal intima, initial lesions, fatty streaks, or fibroatheroma was observed between desialylated and sialylated IDL.

### 3.3. Binding of Sialylated and Desialylated LDL to Collagen

Table 3 lists the results for LDL. The binding to collagen from the PG-rich layer of the uninvolved intima appeared to be similar among sialylated LDL, IDL, and VLDL.

1.4- to 1.8-fold less sialylated LDL bound to collagen from the PG-rich layer of initial lesion, fatty streak, and fibroatheroma compared with the PG-rich layer of uninvolved intima.

The binding capacity for sialylated LDL did not differ significantly among collagens from the intimal musculoelastic layer and the media of all tested tissue types.

Desialylated and sialylated LDL showed similar binding to each of the collagens isolated from uninvolved regions, initial lesions, and fatty streaks. However, with fibroatheroma, we found a better binding of desialylated LDL: its amount bound to collagen from the PG-rich and musculoelastic layers was 1.2- and 1.4-fold greater, respectively, than the amount of bound sialylated LDL.

### 3.4. Binding of Sialylated and Desialylated HDL to Collagen

As seen from the data presented in Table 4, there was no statistically significant difference in binding of sialylated HDL to the collagens isolated from both the intimal layers and media taken from areas of uninvolved tissue, initial lesions, and the fatty streak. When collagen was isolated from the intimal layers of fibroatheroma and the media underlying the fibroatheroma, it bound 1.4- to 1.8-fold more sialylated HDL compared with collagen of normal tissue.

Desialylated HDL bound to collagen from the intimal PG-rich layer of uninvolved areas, initial lesions, and the fatty streak better than sialylated HDL. On the contrary, the binding of desialylated versus sialylated HDL was 2-fold lower when collagen from the media underlying fibroatheroma was used. No significant difference was seen between sialylated and desialylated HDL with the respect to their binding to collagen from the musculoelastic layer of nonatheroslerotic or atherosclerotic intima.

### 3.5. The Influence of Sialic Acid Removal on Lipoprotein Binding to Collagen

Neuraminidase was used to remove sialic acid from sialylated lipoprotein. Neuraminidase treatment reduced the sialic acid content of lipoproteins by 60–73% compared with untreated controls. A comparative analysis between sialylated and chemically desialylated samples was performed by measuring lipoprotein binding to collagen from the intimal PG-rich layer of uninvolved areas and fibroatheroma. The results are presented in Table 5.

Treatment of native (sialylated) VLDL, IDL, and LDL had no effect on their binding to collagen from the intimal PG-rich layer when the latter was taken from uninvolved areas. However, the binding of neuraminidase-treated apoB-containing lipoproteins to collagen from the intimal PG-rich layer of fibroatheroma was found to be 1.8- to 3.5-fold higher than that of native lipoproteins. Thus, a loss of sialic acid stimulated binding of apoB-containing lipoproteins to collagen from lesioned regions, but not from uninvolved tissue.

No difference in binding to collagen isolated either from uninvolved intima or from fibroatheromas appeared between the treated and untreated HDL.

## 4. Discussion

The present results show that circulating lipoproteins can bind to collagens isolated from the PG-rich and musculoelastic layers of the intima and the media of uninvolved and atherosclerotic regions of the human aorta. The novelty of findings evolves from comparative evaluation of the binding of sialylated (native) and desialylated (modified) lipoproteins (VLDL, LDL, LDL, and HDL) to collagen. We have demonstrated that the binding of sialylated (native) apoB-containing lipoproteins was best to collagen from the intimal PG-rich layer. However, the binding of sialylated apoB-containing lipoproteins to collagen from PG-rich layer of fatty streak and fibroatheroma was 1.4- and 3.1-fold lower, respectively, in comparison with normal intima. Desialylated (modified) VLDL versus sialylated one exhibited a greater binding (1.4- to 3.0-fold) to all the collagen preparations examined; desialylated (modified) IDL and LDL also showed a higher binding than sialylated ones when collagen from the intimal layers of fibroatheroma was used. Binding of desialylated (modified) HDL to collagen from the intimal PG-rich layer of normal tissue, initial lesion, and fatty streak was 1.2- to 2.0-fold higher compared with sialylated (native) HDL. These findings clearly demonstrate that at least two variables (lipoprotein modification and the type of atherosclerotic lesion) play the decisive role in the efficacy of lipoprotein binding to collagen.

Of the two collagens of the uninvolved intima-media, the one with the highest binding capacity for apoB-containing lipoproteins was collagen from the intimal PG-rich layer. In the case of an initial lesion, collagen from this layer also showed a higher binding activity, but only for VLDL and IDL. These data indicate that, in the human aortic intima, collagen of the PG-rich layer and the musculoelastic layer are different in their lipoprotein-binding properties. Unfortunately, we failed to find published information on the collagen composition of the distinct layers of the human arterial intima.

VLDL, IDL, and LDL bound less to collagen from the intimal PG-rich layer of the fatty streak and fibroatheroma than to collagen from the respective layer of uninvolved intima. In addition, LDL revealed a lower binding to collagen from this layer of an initial lesion compared with normal intima. In the human arterial wall, the alterations related to atherosclerosis are mainly confined to the PG-rich layer of the intima. It is not surprising, therefore, that, in our study, collagen isolated just from this layer exhibited varying binding activity for apoB-containing lipoproteins, depending on which tissue, normal or lesioned, was taken and the stage of lesion development.

A decreased binding of apoB-containing lipoproteins to collagen from the atherosclerotic intima may reflect disease-related changes in collagen composition of the aortic tissue. It was shown earlier that the main collagens in human aortas were type I and type III [31–35]. Data on the relative content of these collagen phenotypes are contradictory. On one hand, McCullagh and Balian reported that collagen type I, which was low in comparison to type III in normal aortas, exceeded type III in atherosclerotic aortas [33]. On the other hand, some other investigators showed that in uninvolved, as well as atherosclerotic, aortas, most of the collagen was of type I [15, 36–38]. These discrepancies, probably, may be attributed to variations in material and/or procedures for the isolation of collagen. For example, type III collagen is known to be extracted by pepsin more readily than type I collagen [39]. However, despite contradictions in regard to the percentage of collagen types in unaffected human aortic intima, all authors note that type I collagen increases in atherosclerosis, whereas type III collagen decreases [15, 35, 37, 40]. As for the total collagen, its level is by far elevated in atherosclerotic lesions [15, 35].

Human aortas have been shown to contain, besides collagen types I and III, also collagen type V [36, 37, 41]. Morton and Barnes [37] and Murata et al. [15] found that the amount of collagen type V in human aortas was comparable with that of collagen type III and constituted 15–20% of collagen type I. In addition, it was demonstrated that the share of type V collagen was 2-fold higher in atherosclerotic aortas than in normal aortas [15, 37]. The human aortas also contain small amounts of collagen types IV and VI [15, 31, 42]. Murata et al. found a considerable increase in type IV collagen in atherosclerotic lesions [15]: its content was shown to be 6- to 7-fold higher compared with type VI.

Although there are variations, a conclusion from the discussed data is that the relative contents of collagen types IV and V are increased in atherosclerotic aorta, while those of collagen types I and III are decreased. Using a radioisotope method, Greilberger J. et al. found that the binding activities for oxidized LDL followed the order: type I > type V and type III > type IV collagen [20]. Therefore, the collagen types that decrease in atherosclerotic lesions are those which can bind LDL well, and the types that increase have a low binding activity for LDL. In light of these considerations, and taking into account the fact that the collagen preparations, used in our study, included total collagen, we may suppose that disease-related changes in the collagen composition account for a decreased binding of apoB-containing lipoproteins to collagen from the PG-rich layer of the atherosclerotic intima.

The pattern of HDL binding to various collagen preparations differed from the pattern for apoB-containing lipoproteins. The amount of bound HDL was similar in all three collagen preparations from the uninvolved intima-media, and the level of binding did not change when initial lesions or fatty streaks, instead of uninvolved regions, were used for the isolation of collagen. Binding, however, was increased when collagen from the PG-rich and musculoelastic layers of fibroatheroma or the media underlying fibroatheroma was used.

Compared to sialylated VLDL, *in vivo* desialylated VLDL exhibited better binding to all types of collagen preparations examined. *In vivo* desialylated IDL and LDL bound to collagen to a greater extent than sialylated ones if the collagen was isolated from the PG-rich or musculoelastic layers of fibroatheroma. These data imply that the difference in binding to collagen between VLDL and other apoB-containing lipoproteins is not associated solely with sialic acid level. This is confirmed by the results obtained with chemically desialylated lipoproteins. Partial desialylation of native, sialylated lipoproteins was achieved by neuraminidase treatment. The apoB-containing lipoproteins devoid of a part of sialic acid showed an increased ability to bind to collagen from the PG-rich layer of fibroatheroma, but not of uninvolved intima. Thus, sialic acid removal and a concomitant change in the lipoprotein surface charge do not affect the binding of lipoproteins, including VLDL, to collagen of normal intimal tissue. We have previously shown that desialylated VLDL compared with sialylated VLDL has markedly reduced levels of neutral lipids and phospholipids (Tertov, in preparation). Therefore, we may assume that just a loss of lipids favors the binding of desialylated VLDL to collagen of normal aortic tissue. This assumption is in agreement with the current observation that the binding of sialylated apoB-containing lipoproteins to collagen from the PG-rich layer of normal intima decreases in the direction from LDL to IDL to VLDL, that is, as the lipid/protein ratio increases. It is thus possible that this relatively low level of sialylated VLDL binding results from masking the collagen-binding loci by lipids.

IDL and LDL showed an increase in their binding to collagen from the intimal PG-rich layer of fibroatheroma after neuraminidase treatment. As mentioned above, a fibroatheroma is characterized by significant changes in the collagen composition and collagen properties as compared to the unaffected intima. Apparently, an efficient interaction between neuraminidase-treated apoB-containing lipoproteins and collagen of fibroatheroma may be related largely to these changes. Thus, a fall in the sialic acid content may be one of the factors that promote binding desialylated apoB-containing lipoproteins to collagen of atherosclerotic lesions. One more factor that may contribute to the binding of desialylated lipoproteins to collagen is their increased electronegative surface charge.

As we have earlier shown, desialylated LDL, isolated from the total LDL fraction, has an increased electronegative charge relative to sialylated LDL. One may expect that a loss of sialic acid makes the surface charge of a lipoprotein particle more positive. However, as follows from our previous studies, desialylated LDL may be referred to as multiple-modified lipoproteins since they differ from native lipoproteins by a number of alterations, including the modified lysine residues and modified tertiary structure of apolipoprotein. These modifications increase the negative charge of the apolipoprotein surface. The importance of negative surface charge in the interaction of LDL with collagen was indicated earlier by observations that collagen bound oxidized LDL, whose negative charge is increased, more efficiently. For example, Hoover et al. [18], by using a gel diffusion technique, demonstrated that oxidatively modified ^125^I-LDL bound better to collagen than nonoxidized ^125^I-LDL, and the amount of binding depended on the extent of modification. Based on these results, the authors have assumed that a net negative surface charge of lipoproteins is the determining factor for their binding to collagen. This assumption was later supported by others. Greilberger et al. [20] showed that HDL_3_, native and oxidized with CuSO_4_, could bind to collagen types I and III and that 2h-oxidized HDL_3_, which had the highest negative charge had the highest binding activity. Chen et al. [43] also showed that oxidized LDL interacted more efficiently with collagen compared to native LDL, and increasing the negative charge in oxidized LDL was associated with progressively greater increases in binding to collagen. The enhanced binding of desialylated lipoproteins to collagen may be explained in part by the presence of a greater negative charge on the surface of desialylated lipoprotein particle.

The influence of neuraminidase treatment of HDL on binding to collagen from the PG-rich layer was observed neither when this layer was taken from uninvolved intima nor when it was taken from fibroatheroma. Hence, sialic acid seems to be unessential for HDL binding to collagen. A greater binding of desialylated HDL versus sialylated HDL, seen with collagen from the PG-rich layer of normal intima, initial lesions, and fatty streaks, is probably due to an increase in the electronegative surface charge in desialylated lipoprotein, but not to a loss of sialic acid.

The present study has demonstrated that collagen from various layers of unaffected and atherosclerotic human aortic intima-media is capable of binding different fractions of human blood lipoproteins. *In vivo*, this binding may contribute to extracellular lipid accumulation. Further studies on the interaction of native and modified lipoproteins with different types of collagen from human aortas should establish mechanisms of this interaction and ways to regulate this process.

## Figures and Tables

**Table 1:** Binding of sialylated and desialylated VLDL to collagen isolated from two layers of the intima and the media of uninvolved and atherosclerotic areas of human aorta.

Lipoprotein	Collagen-bound apoB, pg/well		
	PG-rich layer	Musculoelastic layer	Media
Uninvolved area			

Sialylated VLDL	414 ± 37	143 ± 10	150 ± 4*
Desialylated VLDL	650 ± 58*	299 ± 20*	328 ± 4

Initial lesion			

Sialylated VLDL	435 ± 10	126 ± 10	120 ± 12*
Desialylated VLDL	598 ± 18*	310 ± 20*	304 ± 15

Fatty streak			

Sialylated VLDL	135 ± 11^&^	113 ± 10	130 ± 7
Desialylated VLDL	233 ± 10^∗&^	339 ± 10*	332 ± 8*

Fibroatheroma			

Sialylated VLDL	180 ± 12^&^	127 ± 3	165 ± 4
Desialylated VLDL	383 ± 20^∗&^	336 ± 30*	440 ± 9*

Each well of 96-well microtiter plates was coated with collagen and washed as described in Section 2. The binding of VLDL (10 *μ*g/mL) was performed at 37°C for 2 h. Values are the means ± S.E.M (*n* = 4); *significant difference from sialylated VLDL (*P* < 0.05); ^&^significant difference from uninvolved region (*P* < 0.05).

**Table 2:** Binding of sialylated and desialylated IDL to collagen isolated from two layers of the intima and the media of uninvolved and atherosclerotic areas of human aorta.

Lipoprotein	Collagen-bound apoB, pg/well		
	PG-rich layer	Musculoelastic layer	Media
Uninvolved area			

Sialylated IDL	512 ± 21	325 ± 27	385 ± 27
Desialylated IDL	559 ± 61	393 ± 12	418 ± 19

Initial lesion			

Sialylated IDL	547 ± 27	270 ± 32	237 ± 19
Desialylated IDL	554 ± 18	287 ± 19	229 ± 7

Fatty streak			

Sialylated IDL	214 ± 4^&^	236 ± 6	234 ± 14
Desialylated IDL	243 ± 17^&^	296 ± 13	258 ± 14

Fibroatheroma			

Sialylated IDL	331 ± 18^&^	187 ± 16	260 ± 18
Desialylated IDL	419 ± 19^∗&^	238 ± 2*	276 ± 12

Each well of 96-well microtiter plates was coated with collagen and washed as described in Section 2. The binding of IDL (10 *μ*g/mL) was performed at 37^o^C for 2 h. Values are the means ± S.E.M (*n* = 4); *significant difference from sialylated IDL (*P* < 0.05); ^&^significant difference from uninvolved region (*P* < 0.05).

**Table 3:** Binding of sialylated and desialylated LDL to collagen isolated from two layers of the intima and the media of uninvolved and atherosclerotic areas of human aorta.

Lipoprotein	Collagen-bound apoB, pg/well		
	PG-rich layer	Musculoelastic layer	Media

Uninvolved area			

Sialylated LDL	507 ± 27	331 ± 8	408 ± 13
Desialylated LDL	416 ± 47	350 ± 10	353 ± 15

Initial lesion			

Sialylated LDL	363 ± 18^&^	373 ± 12	426 ± 14
Desialylated LDL	374 ± 14	374 ± 7	517 ± 7

Fatty streak			

Sialylated LDL	277 ± 5^&^	332 ± 26	356 ± 17
Desialylated LDL	290 ± 5	317 ± 17	403 ± 12

Fibroatheroma			

Sialylated LDL	460 ± 11^&^	365 ± 13	458 ± 15
Desialylated LDL	539 ± 16*	508 ± 13*	527 ± 15

Each well of 96-well microtiter plates was coated with collagen and washed as described in Section 2. The binding of LDL (10 *μ*g/mL) was performed at 37°C for 2 h. Values are the means ± S.E.M (*n* = 4); *significant difference from sialylated LDL (*P* < 0.05); ^&^significant difference from uninvolved region (*P* < 0.05).

**Table 4:** Binding of sialylated and desialylated HDL to collagen isolated from two layers of the intima and the media of uninvolved and atherosclerotic areas of human aorta.

Lipoprotein	Collagen-bound apoA1, pg/well		
	PG-rich layer	Musculoelastic layer	Media
Uninvolved area			

Sialylated HDL	279 ± 4	312 ± 12	274 ± 50
Desialylated HDL	320 ± 3*	319 ± 3	276 ± 13

Initial lesion			

Sialylated HDL	256 ± 15	299 ± 14	369 ± 16
Desialylated HDL	524 ± 34^&∗^	280 ± 9	378 ± 13

Fatty streak			

Sialylated HDL	256 ± 27	313 ± 17	320 ± 43
Desialylated HDL	431 ± 26^&∗^	290 ± 17	310 ± 16

Fibroatheroma			

Sialylated HDL	382 ± 36^&^	500 ± 18^&^	497 ± 15^&^
Desialylated HDL	290 ± 12	508 ± 14^&^	242 ± 17*

Each well of 96-well microtiter plates was coated with collagen and washed as described in Section 2. The binding of HDL (10 *μ*g/mL) was performed at 37°C for 2 h. Values are the means ± S.E.M (*n* = 4); *significant difference from sialylated HDL (*P* < 0.05); ^&^significant difference from uninvolved region (*P* < 0.05).

**Table 5:** Effect of neuraminidase treatment on lipoprotein sialic acid content and lipoprotein binding to collagen.

Lipoprotein	Sialic acid content, % of control	Binding to collagen from the PG-rich layer of normal intima, % of control	Binding to collagen from the PG-rich layer of fibroatheroma, % of control
VLDL	40 ± 3*	107 ± 6	178 ± 11*
IDL	28 ± 3*	117 ± 9	214 ± 12*
LDL	27 ± 2*	108 ± 10	347 ± 21*
HDL	36 ± 3*	114 ± 8	117 ± 9

Sialylated lipoproteins were treated with 0.02 U/mL neuraminidase from *Arthrobacter ureafaciens* at 37°C for 6 h. Control lipoproteins were incubated in the absence of neuraminidase. Lipoproteins were then reisolated by ultracentrifugation, and microtiter well binding assay was performed as described in Section 2. Values are the means ± S.E.M (*n* = 4); *significant difference from control (*P* < 0.05).

## References

[1] Noma A, Takahashi T, Wada T (1981). Elastin-lipid interaction in the arterial wall—part 2. In vitro binding of lipoprotein-lipids to arterial elastin and the inhibitory effect of high density lipoproteins on the process. *Atherosclerosis*.

[2] Tokita K, Kanno K, Ikeda K (1977). Elastin sub-fraction as binding site for lipids. *Atherosclerosis*.

[3] Camejo G, Lopez A, Lopez F, Quinones J (1985). Interaction of low density lipoproteins with arterial proteoglycans. The role of charge and sialic acid content. *Atherosclerosis*.

[4] Srinivasan SR, Vijaayagopal P, Radhakrishnamurthu B, Berenson GS (1989). Low-density lipoprotein binding affinity of arterial wall proteoglycans: characteristics of chondroitin sulfate proteoglycan subfraction. *Biochimica et Biophysica Acta*.

[5] Smith EB (1965). The influence of age and atherosclerosis on the chemistry of aortic intima. *Journal of Atherosclerosis Research*.

[6] Grant RA (1967). Content and distribution of aortic collagen, elastin and carbohydrate in different species. *Journal of Atherosclerosis Research*.

[7] Ross R, Glomset JA (1976). The pathogenesis of atherosclerosis. *New England Journal of Medicine*.

[8] Balleisen L, Gay S, Marx R, Kuehn K (1975). Comparative investigation on the influence of human bovine collagen types I, II and III on the aggregation of human platelets. *Klinische Wochenschrift*.

[9] Barnes MJ, Gordon JL, MacIntyre DE (1976). Platelet aggregating activity of type I and type III collagens from human aorta and chicken skin. *Biochemical Journal*.

[10] Levene CI, Poole JC (1962). The collagen content of the normal and atherosclerotic human aortic intima. *British Journal of Experimental Pathology*.

[11] McCullagh KA, Balian G (1975). Collagen characterisation and cell transformation in human atherosclerosis. *Nature*.

[12] Bertelsen S (1968). Chemical studies on the arterial wall in relation to atherosclerosis. *Annals of the New York Academy of Sciences*.

[13] McCullagh KG, Duance VC, Bishop KA (1980). The distribution of collagen types I, III and V (AB) in normal and atherosclerotic human aorta. *Journal of Pathology*.

[14] Morton LF, Barnes MJ (1982). Collagen polymorphism in the normal and diseased blood vessel wall. Investigation of collagens types I, III and V. *Atherosclerosis*.

[15] Murata K, Motayama T, Kotake C (1986). Collagen types in various layers of the human aorta and their changes with the atherosclerotic process. *Atherosclerosis*.

[16] Hanson AN, Bentley JP (1983). Quantitation of type I to Type III collagen ratios in small samples of human tendon, blood vessels, and atherosclerotic plaque. *Analytical Biochemistry*.

[17] Le Lous M, Boudin D, Salmon S, Polonovski J (1982). The affinity of type I collagen for lipid in vitro. *Biochimica et Biophysica Acta*.

[18] Hoover GA, McCormick S, Kalant N (1988). Interaction of native and cell-modified low density lipoprotein with collagen gel. *Arteriosclerosis*.

[19] Jimi S, Sakata N, Matunaga A, Takebayashi S (1994). Low density lipoproteins bind more to type I and III collagens by negative charge-dependent mechanisms than to type IV and V collagens. *Atherosclerosis*.

[20] Greilberger J, Schmut O, Jurgens G (1997). In vitro interactions of oxidatively modified LDL with type I, II, III, IV, and V collagen, laminin, fibronection, and poly-D-Lysine. *Arteriosclerosis, Thrombosis, and Vascular Biology*.

[21] Orekhov AN, Tertov VV, Mukhin DN, Mikhailenko IA (1989). Modification of low density lipoprotein by desialylation causes lipid accumulation in cultured cells: discovery of desialylated lipoprotein with altered cellular metabolism in the blood of atherosclerotic patients. *Biochemical and Biophysical Research Communications*.

[22] Orekhov AN, Tertov VV, Mukhin DN (1991). Desialylated low density lipoprotein—naturally occurring modified lipoprotein with atherogenic potency. *Atherosclerosis*.

[23] Tertov VV, Orekhov AN, Martsenyuk ON, Perova NV, Smirnov VN (1989). Low-density lipoproteins isolated from the blood of patients with coronary heart disease induce the accumulation of lipids in human aortic cells. *Experimental and Molecular Pathology*.

[24] Tertov VV, Orekhov AN, Sobenin IA, Morrisett JD, Gotto AM, Guevara JG (1993). Carbohydrate composition of protein and lipid components in sialic acid- rich and -poor low density lipoproteins from subjects with and without coronary artery disease. *Journal of Lipid Research*.

[25] Stary HC (1992). Composition and classification of human atherosclerotic lesions. *Virchows Archiv*.

[26] Orekhov AN, Andreeva ER, Tertov VV, Krushinsky AV (1984). Dissociated cells from different layers of adult human aortic wall. *Acta Anatomica*.

[27] Lansing AI, Rosenthal TB, Alex M, Dempsy EW (1952). The structure and chemical characterization of elastic fibers as revealed by elastase and by electron microscopy. *The Anatomical record*.

[28] Bilheimer DW, Eisenberg S, Levy RI (1972). The metabolism of very low density lipoprotein proteins I. Preliminary in vitro and in vivo observations. *Biochimica et Biophysica Acta*.

[29] Markwell MAK (1982). A new solid-state reagent to iodinate proteins. I. Conditions for the efficient labelling of antiserum. *Analytical Biochemistry*.

[30] Dixon WJ, Brown MB (1977). *Biomedical Computer Programs*.

[31] Trelstad RL (1974). Human aorta collagens: evidence for three distinct species. *Biochemical and Biophysical Research Communications*.

[32] Chung E, Miller EJ (1974). Collagen polymorphism: characterization of molecules with the chain composition [*α*1(III)]3 in human tissues. *Science*.

[33] McCullagh KA, Balian G (1975). Collagen characterisation and cell transformation in human atherosclerosis. *Nature*.

[34] Barnes MJ, Gordon JL, MacIntyre DE (1976). Platelet aggregating activity of type I and type III collagens from human aorta and chicken skin. *Biochemical Journal*.

[35] Barnes MJ (1985). Collagens in atherosclerosis. *Collagen and Related Research*.

[36] Ooshima A (1981). Collagen B chain: increased proportion in human atherosclerosis. *Science*.

[37] Morton LF, Barnes MJ (1982). Collagen polymorphism in the normal and diseased blood vessel wall. Investigation of collagens types I, III and V. *Atherosclerosis*.

[38] Hanson AN, Bentley JP (1983). Quantitation of type I to Type III collagen ratios in small samples of human tendon, blood vessels, and atherosclerotic plaque. *Analytical Biochemistry*.

[39] Szymanowicz AG, Bellon G, Laurain-Guillaume G (1982). An evaluation by sequential extraction of the proportions of collagen types from medium sized arteries. *Artery*.

[40] McCullagh KG (1983). Increased type I collagen in human atherosclerotic plaque. *Atherosclerosis*.

[41] Chung E, Rhodes RK, Miller EJ (1976). Isolation of three collagenous components of probable basement membrane origin from several tissues. *Biochemical and Biophysical Research Communications*.

[42] Mayne R, Zettergren JG, Mayne PM, Bedwell NW (1980). Isolation and partial characterization of basement membrane-like collagens from bovine thoracic aorta. *Artery*.

[43] Chen CH, Henry PD (1997). Atherosclerosis as a microvascular disease: impaired angiogenesis mediated by suppressed basic fibroblast growth factor expression. *Proceedings of the Association of American Physicians*.

